# Glass in the Foot Fifteen Years

**Published:** 1862-10

**Authors:** Geo. Couse

**Affiliations:** Strathray


ART. IV.—*Glass in the foot fifteen years.*

STRATHRAY, September 17, 1862.

Dr. Miner—Dear Sir:

Enclosed I send you a bit of glass one inch long by about one-half an inch wide at the base, terminating in a sharp point, which I took from Mrs. Couse's foot two years ago this month, which had been in the bottom of the foot for over fifteen years. The little boys had thrown a play-ball through a window opening in her bedroom; the fragments of glass fell upon the carpet, and upon retiring to bed she walked upon them; some three or four pieces were taken out at the time. After that, at times, she felt sharp pains in the foot, but no great inconvenience ever arose from it, could walk and dance as well as other young ladies. At the time my little girl was ill she was a great deal upon her feet, and then for the first, for about a fortnight, it had made her very lame. The fragment of glass passed in about the head of the metatarsal bone of the great toe, passed across the sole, and made its appearance about an inch back from the head of the metatarsal bone of the little toe, the base or broad end presenting. I extracted it with ease.

I can vouch for the truth of the above, and am aware, or believe that in your extensive field you must have seen similar cases. Hoping to hear from you at your earliest convenience, I subscribe myself

Truly yours, GEO. COUSE.

